# MicroRNAs as Regulators of Radiation-Induced Oxidative Stress

**DOI:** 10.3390/cimb46070423

**Published:** 2024-07-06

**Authors:** Branislav Kura, Patricia Pavelkova, Barbora Kalocayova, Margita Pobijakova, Jan Slezak

**Affiliations:** 1 Centre of Experimental Medicine, Slovak Academy of Sciences, Dubravska Cesta 9, 841 04 Bratislava, Slovakia; patricia.pavelkova@savba.sk (P.P.); barbora.kalocayova@savba.sk (B.K.); jan.slezak@savba.sk (J.S.); 2 Department of Pharmacology and Toxicology, Faculty of Pharmacy, Comenius University, 832 32 Bratislava, Slovakia; 3 Department of Radiation Oncology, Bory Hospital–Penta Hospitals, 841 03 Bratislava, Slovakia; margita.pobijakova@pentahospitals.com; 4 Radiological Science, Faculty of Nursing and Medical Professional Studies, Slovak Medical University, 831 01 Bratislava, Slovakia

**Keywords:** apoptosis, cell fate, microRNA, oxidative stress, radiation, ROS

## Abstract

microRNAs (miRNAs) represent small RNA molecules involved in the regulation of gene expression. They are implicated in the regulation of diverse cellular processes ranging from cellular homeostasis to stress responses. Unintended irradiation of the cells and tissues, e.g., during medical uses, induces various pathological conditions, including oxidative stress. miRNAs may regulate the expression of transcription factors (e.g., nuclear factor erythroid 2 related factor 2 (Nrf2), nuclear factor kappa B (NF-κB), tumor suppressor protein p53) and other redox-sensitive genes (e.g., mitogen-activated protein kinase (MAPKs), sirtuins (SIRTs)), which trigger and modulate cellular redox signaling. During irradiation, miRNAs mainly act with reactive oxygen species (ROS) to regulate the cell fate. Depending on the pathway involved and the extent of oxidative stress, this may lead to cell survival or cell death. In the context of radiation-induced oxidative stress, miRNA-21 and miRNA-34a are among the best-studied miRNAs. miRNA-21 has been shown to directly target superoxide dismutase (SOD), or NF-κB, whereas miRNA-34a is a direct regulator of NADPH oxidase (NOX), SIRT1, or p53. Understanding the mechanisms underlying radiation-induced injury including the involvement of redox-responsive miRNAs may help to develop novel approaches for modulating the cellular response to radiation exposure.

## 1. Introduction

Oxidative stress is a result of the imbalance between the intracellular formation of reactive species, such as reactive oxygen species (ROS) and reactive nitrogen species (RNS), and the ability of a cell to eliminate them. The vast majority of cellular ROS originate from mitochondria due to oxidative phosphorylation. They are also produced by plasma membrane enzymes (e.g., NADPH oxidase (NOX)) or various cytosolic enzymes (e.g., cyclooxygenase (COX)) [1]. Although ROS and RNS act as signaling molecules under physiological concentrations, excessive amounts of ROS and RNS exert cellular damage through their oxidative effect on proteins, lipids, and DNA [2].

Environmental oxidative stress derives from different artificial and natural sources such as cigarette smoke, traffic exhaust emissions, solar radiation, nutrition, radiotherapy, cosmic rays, or industrial pollution [3]. The absorption of ionizing radiation by living cells can directly disrupt atomic structures or acts indirectly through radiolysis of water, thereby generating mainly hydroxyl radicals (^•^OH). Both the direct and indirect effects of radiation initiate a series of signaling events that may repair cell damage or lead to cell death [4].

microRNAs (miRNAs) are small (~22 nucleotides) non-coding RNA molecules that negatively regulate gene expression at the post-transcriptional level by preventing the translation of messenger RNA (mRNA) or by accelerating mRNA degradation. This process is highly complex, because each miRNA is able to act upon several different target genes, whilst one target gene can be regulated by many different miRNAs [5]. In mammals, miRNAs are predicted to control the activity of ~30% of all protein-coding genes [6].

**Figure 1:**
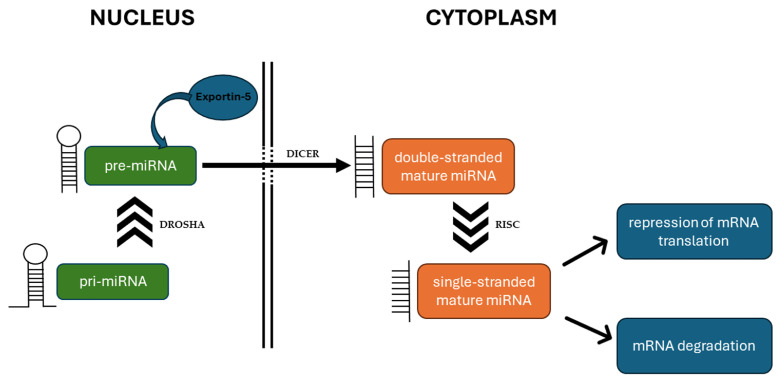
The schema of microRNA (miRNA) biogenesis. In the nucleus, pri-miRNA is cleaved by the enzyme DROSHA into pre-miRNA and exported to the cytoplasm by exportin-5. In the cytoplasm, pre-miRNA is processed by the enzyme DICER into the mature miRNA duplex. The duplex is loaded into the RNA-induced silencing complex (RISC), where it is unwound to a final single-stranded form. The RISC/miRNA complex then binds to target mRNAs and represses their translation or promotes their degradation.

miRNA biogenesis starts with the formation of a primary nuclear transcript (pri-miRNA). The pri-miRNA is cleaved by the RNase III enzyme DROSHA into a precursor form (pre-miRNA) and exported into the cytoplasm. The pre-miRNA enters into a complex with a second RNase III enzyme, DICER, where it is processed into the mature form. Subsequently, the RNA duplex is transferred into the RNA-induced silencing complex (RISC). Here, the double-stranded miRNA is unwound to produce a single-stranded miRNA that is ready to bind with a complementary sequence in the target mRNAs [7] (Figure 1).

Emerging evidence confirms that miRNAs are involved in the regulation of radiation-induced cellular processes. miRNAs regulate the cellular response to radiation by participating in multiple pathways involved in DNA repair, cell cycle checkpoints, apoptosis, autophagy, and oxidative stress [8]. An interplay between miRNAs and redox signaling has been reported [9]. miRNAs may regulate the expression of redox sensors and other ROS modulators, such as transcription factors (e.g., nuclear factor erythroid 2 related factor 2 (Nrf2), tumor suppressor protein p53, nuclear factor kappa B (NF-κB)) and other redox-sensitive genes (e.g., mitogen-activated protein kinase (MAPK), sirtuin (SIRT)), which trigger and modulate cellular redox signaling. This can be achieved via direct targeting of mRNA of these genes or indirectly via targeting mRNAs encoding proteins that control the level and activity of redox-sensitive genes [10].

The aim of this review is to elucidate the involvement of miRNAs in the regulation of oxidative stress induced by irradiation.

## 2. Radiation and Oxidative Stress

Oxidative stress is characterized by the excessive generation of ROS and RNS overwhelming cellular antioxidant defenses. Free radicals and non-radical species like superoxide anions (O_2_^•−^), hydrogen peroxide (H_2_O_2_), and ^•^OH radicals play pivotal roles in cellular signaling and homeostasis while also causing oxidative damage to biomolecules if produced in excess [11,12]. ROS are highly reactive and can interact with biological macromolecules, such as DNA, proteins, and lipids, thereby changing the functions of these macromolecules [13].

Cells maintain ROS homeostasis by reducing ROS production and triggering specific antioxidant mechanisms to neutralize ROS or mitigate oxidative stress. Antioxidant enzymes include superoxide dismutase (SOD), catalase (CAT), peroxiredoxins (PRDXs), thioredoxins (TXNs), glutathione peroxidase (GPx), and heme oxygenase (HO). In this process, SOD converts O_2_^•−^ to O_2_ or H_2_O_2_. Subsequently, CAT and GPx convert H_2_O_2_ to H_2_O and O_2_ [14].

ROS may be generated by exogenous or endogenous sources. Exogenous sources encompass ionizing and non-ionizing radiation, drugs, pollutants, food, ultrasound, xenobiotics, and toxins [15]. Endogenous ROS sources are cellular organelles with high oxygen consumption such as mitochondria, peroxisomes, and endoplasmic reticulum [16]. In human cells, more than 50 enzymes produce O_2_^•−^ and H_2_O_2_, with mitochondria and NOX being the principal sources [17].

**Figure 2:**
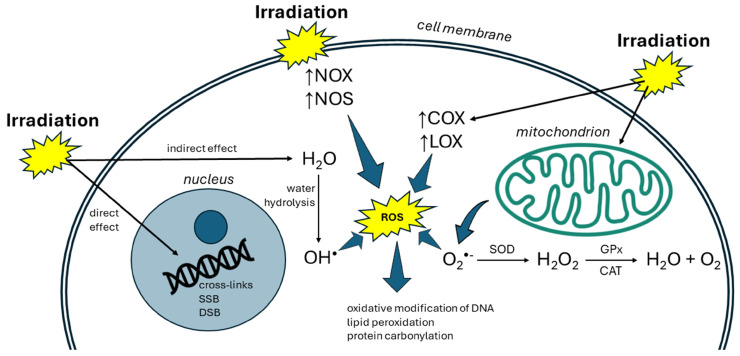
The effect of radiation on cells. Radiation can affect cells via direct and indirect mechanisms. The direct effect includes ionization of biomolecules and disruption of bonds. Indirectly, radiation acts via the radiolysis of water, thereby producing reactive oxygen species (ROS), mainly hydroxyl radicals (^•^OH). ROS subsequently damage biomolecules like DNA, lipids, and proteins. Disruption of the cellular redox balance may lead to cell death. superoxide (O_2_^•−^), superoxide dismutase (SOD), catalase (CAT), glutathione peroxidase (GPx), single-strand breaks (SSB), double-strand breaks (DSB), cyclooxygenase (COX), lipoxygenase (LOX), NADPH oxidase (NOX), and nitric oxide synthase (NOS).

Ionizing radiation, such as X-rays, neutrons, and α-, β-, and γ-rays, disrupts cell redox homeostasis and leads to oxidative stress that may result in cell death [18]. The deleterious effects of ionizing radiation can manifest through direct and indirect mechanisms (Figure 2). In addition to direct ionization of critical biomolecules, ionizing radiation can induce cellular damage indirectly via the production of ROS [19]. Almost 80% of tissues and cells are composed of water. Therefore, most of the radiation damage is caused by the generation of ROS stemming from the radiolysis of water, mainly ^•^OH radicals [20]. Among the radicals, ^•^OH is the most reactive and is able to react with almost any tissue constituents directly, thereby causing more effective cellular damage than any other ROS [21].

DNA damage induced by irradiation ranges from base modifications and single-strand breaks to double-strand breaks and cross-links. This can lead to disruption of genomic integrity and to the initiation of mutagenesis [22]. Oxidative stress causes harm to both mitochondrial and nuclear DNA. 8-hydroxy-2′-deoxyguanosine (8-OHdG) may be used as an indicator of radiation DNA damage [23]. Lipid peroxidation, which occurs because of radiation-induced oxidative stress, leads to the formation of reactive lipid derivatives that can harm other biomolecules. 4-hydroxy-2-nonenal (4-HNE) and malondialdehyde (MDA) are examples of the final products of lipid peroxidation, utilized as indicators of the degree of lipid oxidative damage [24]. Oxidative alterations to the structure of proteins occur as a consequence of radiation damage, which results in changes to their spatial conformation, hindered degradation, and the formation of modified protein products like protein carbonyl derivatives [25].

The mitochondrial electron transport chain is the major site of ROS production in most mammalian cells. The exposure of cells to irradiation stimulates the production of endogenous ROS in mitochondria, leading to mitochondrial dysfunction and programmed cell death [26]. The remaining damaged mitochondria produce and release more ROS inside the cell, which leads to programmed cell death [27]. Free radicals produced by irradiation can upregulate several enzymes including NOX, lipoxygenases (LOXs), nitric oxide synthase (NOS), and COX, thereby contributing to the elevation of oxidative stress [28]. The inhibition of antioxidant enzymes is one of the important effects of ionizing radiation on irradiated cells, which leads to the production and accumulation of ROS [29].

Oxidative alterations may potentially persist months or even years post-irradiation. This prolonged oxidative burden likely arises from continuous ROS/RNS generation via diverse mechanisms, including mitochondrial damage, inflammatory responses, cell death processes, overexpression of ROS-generating enzymes, and inadequate antioxidant defenses [4].

## 3. MiRNAs Affect ROS Production and Elimination

Growing evidence confirms that miRNAs target antioxidant-responsive elements and ROS-related genes, thus affecting cellular redox status. Gene silencing by miRNAs can affect ROS activators and ROS scavengers, leading to a complex interplay between oxidative stress and miRNAs to modulate cellular redox homeostasis [30]. miRNAs can have positive or negative effects on ROS production by controlling genes regulating ROS biogenesis and scavenging. Moreover, ROS co-regulate with miRNAs by modulating their transcription and maturation [31].

The published literature reveals that miRNAs can target ROS generation and modulate antioxidant signaling. In this context, the regulation of different transcription factors (Nrf2, p53, NF-κB) and enzymes (NOX, MAPK, SIRT) by miRNAs has mainly been described. miRNAs targeting endogenous antioxidant enzymes have been reported, as well. Most of the evidence originates from the studies where miRNAs directly targeted relevant genes, binding to their mRNA and subsequently repressing mRNA translation or causing mRNA degradation [30].

The Nrf2 pathway is one of the most important pathways for intracellular protection during oxidative stress. Nrf2 is a transcriptional factor that activates the transcription of genes encoding antioxidant enzymes and other cytoprotective and stress-related genes [32]. The role of miRNAs in regulating Nrf2 signaling is widely recognized. Diverse miRNAs can decrease or activate the Nrf2 pathway through direct targeting of the Nrf2 mRNA and mRNAs encoding proteins that control the level and activity of Nrf2 [33,34]. Upregulation of miRNA-153 in cardiomyocytes results in ROS production and cell apoptosis. It has been demonstrated that miRNA-153 directly targets Nrf2, which inhibits the expression of Nrf2 as well as HO-1 proteins [35]. Similarly, miRNA-140-5p promotes myocardial oxidative stress in doxorubicin-induced cardiotoxicity by directly targeting Nrf2 [36]. On the other hand, miRNA-126 inhibited oxidative stress by inducing Nrf2 after renal ischemic postconditioning in mice [37].

In response to stress, a molecular mechanism facilitated by regulatory Kelch-like ECH-associated protein 1 (Keap1) allows Nrf2 to escape ubiquitination, accumulate within the cell, and translocate to the nucleus, where it can promote its antioxidant transcription program [38]. An increase in Nrf2 expression and activity was observed for miRNAs directly targeting Keap1 mRNA. miRNA-7 represses Keap1 expression by direct targeting its mRNA in human neuroblastoma cells, which activates the Nrf2 pathway [39]. In the human breast cancer cell line MDA-MB-231, miRNA-200a was found to directly target Keap1 mRNA, leading to its degradation [40].

miRNAs may regulate ROS levels by targeting enzymes that are responsible for ROS production, like NOX or NOS [41] (Table 1).

The ability of miRNAs to directly target antioxidant enzymes has been proven in different experimental models [30]. miRNAs can affect ROS levels through the direct activation or inhibition of endogenous antioxidant enzymes including CAT, SOD, GPx, and glutathione reductase (GR) [51] (Table 2).

p53 is a transcription factor that plays key roles in cellular responses to oxidative stress. In response to low oxidative stress, p53 exhibits antioxidant activities to eliminate oxidative stress and ensure cell survival. On the other hand, in response to high levels of oxidative stress, p53 exhibits pro-oxidative activities that further increase the level of stress, leading to cell death [60]. Li et al. [61] found that miRNA-23a promotes apoptosis induced by oxidative stress by directly targeting p53 in cardiomyocytes. Another study revealed that oxidative stress induced the upregulation of miRNA-24 and enhanced lens epithelial cells’ death by directly targeting p53 [62]. Oxidative stress induced by H_2_O_2_ inhibited miRNA-122 expression in human umbilical vein cells (HUVECs). miRNA-122 overexpression attenuated cytotoxic injury in HUVEC cells by increasing cell viability and suppressed cell apoptosis and oxidative stress injury by directly targeting p53 [63].

NF-κB is another transcriptional factor that is regulated in response to oxidative stress. ROS have been documented to have bidirectional effects on NF-κB signaling, causing either activation or suppression depending on the duration and context of exposure. Interestingly, activation of the NF-κB pathway can have both anti- and pro-oxidant effects [64]. Multiple miRNAs have been found to modulate NF-κB activity. miRNA-506 directly targeted and inhibited the expression of the NF-κB p65 subunit and led to the production of ROS and p53-dependent apoptosis in lung cancer cells [65]. On the other hand, miRNA-124 prevented H_2_O_2_-induced oxidative stress and apoptosis in human lens epithelial cells by suppressing the expression of the NF-κB p65 subunit [66]. Similarly, miRNA-26a-5p inhibited oxidative stress and inflammation in diabetic retinopathy progression by inactivating the NF-κB signaling pathway. The authors demonstrated that miRNA-26a-5p inhibited phosphorylation of NF-κB inhibitory protein (IκBα) and NF-κB nuclear translocation [67].

SIRTs represent a family of nicotinamide adenine dinucleotide (NAD)+-dependent histone deacetylases, which have been found to be involved in modulating the levels of ROS as well as antioxidant enzymes in the cell. This is possible through the modulation of key transcription factors such as Nrf2, p53, NF-κB, or Forkhead box O (FOXO). Most of them appear to have antioxidant and/or ROS-suppressive effects [68]. The number of studies oriented toward the identification of miRNAs regulating SIRT under oxidative stress is increasing. SIRT1 has been confirmed as a direct target of several miRNAs (e.g., miRNA-199a, miRNA-34a, miRNA-29b, miRNA-217) [69,70,71,72]. The regulation of other SIRT family members by miRNAs under oxidative stress conditions has also been described [31].

Other molecules associated with ROS are MAPKs, which play the major role in signal transduction from the cell surface to the nucleus and thus regulate several physiological processes. Generally, ROS in a cell are considered to be significant modulators of MAPK pathways, and increased ROS production leads to the activation of MAPKs [73]. It has been demonstrated that miRNA-132 can stimulate the antioxidant system and reduce the apoptosis rate by directly targeting MAPK1 [74]. MAPK activation induced by H_2_O_2_ promoted apoptosis in alveolar epithelial cells by inhibiting miRNA-21-5p expression [75]. miRNA-146a aggravated cognitive impairment by triggering oxidative stress via the upregulation of p38 MAPK [76]. miRNA-30a-5p ameliorated inflammatory responses and oxidative stress induced by spinal cord injury by reducing the levels of phosphorylated p38 MAPK and protein kinase B (AKT) [77].

## 4. Evidence for miRNA Action in Radiation-Induced Oxidative Stress

Many studies detected a changed miRNA expression alongside a changed redox balance after irradiation. In one of the early reports, Simone et al. [78] identified 17 miRNAs with a changed expression following exposure to radiation. Among them, let-7b, let-7e, miRNA-15b, miRNA-21, miRNA-638, miRNA-768-3p, and miRNA-768-5p were also deregulated under other oxidative-stress-induced conditions. Chakraborty et al. [79] examined the consequences of total body irradiation in mice up to 9 days after irradiation. After irradiation, they reported an early escalation of miRNA-155–5p, while a set of other miRNAs was inhibited, such as miRNA-486-5p, miRNA-21-5p, and miRNA-351-5p. miRNA-124 was consistently inhibited across all time phases post-irradiation. This miRNA directly targets NF-κB. They found a significant enrichment of the ROS synthesis network in irradiated mouse plasma. In another study, miRNA-193a-3p was observed to induce apoptosis in both U-251 and HeLa cells after irradiation. The authors also demonstrated that miRNA-193a-3p induced the accumulation of intracellular ROS and DNA damage. The induction of both apoptosis and DNA damage by miRNA-193a-3p was blocked by antioxidant treatment, indicating the crucial role of ROS in the action of miRNA-193a-3p [80].

Different reports support a role for miRNAs in radiation-induced oxidative stress [81,82]. In one, for cells exposed to radiation, the levels of multiple miRNAs that directly target transcripts of enzymes responsible for ROS/RNS production and neutralization were altered; however, the response could be different in different types of cells [41]. A UV-induced redox imbalance could modulate miRNA expression via the activation of transcription factors including NF-κB, p53, hypoxia-inducible factor (HIF)-1α, and Nrf2 [10].

**Figure 3:**
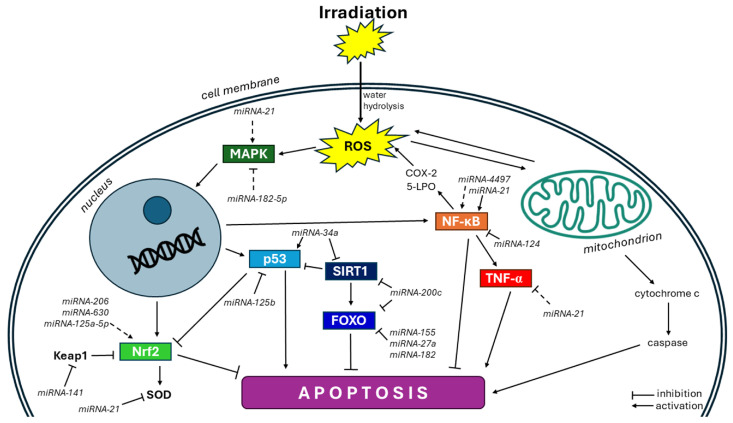
Oxidative-stress-related pathways regulated by microRNAs after irradiation. During irradiation, miRNAs mainly cooperate with ROS to regulate the cell fate. miRNAs have been shown to regulate different transcription factors (nuclear factor erythroid 2 related factor 2 (Nrf2), nuclear factor kappa B (NF-κB), tumor necrosis factor alpha (TNF-α), tumor suppressor protein p53, Forkhead box O (FOXO)), and other redox-sensitive genes (mitogen-activated protein kinase (MAPK), sirtuin (SIRT)) in response to radiation exposure. Kelch-like ECH-associated protein 1 (Keap1), cyclooxygenase 2 (COX-2), 5-lipoxygenase (5-LPO). The solid line means direct targeting by miRNAs, and the dashed line means the indirect effect of miRNAs on the specific gene/pathway.

During irradiation, miRNAs mainly cooperate with ROS to regulate the cell fate. Studies have demonstrated that radiotherapy primarily acts through the intrinsic pathway of apoptosis [83]. Cell survival can be affected by the direct or indirect modification of redox-sensitive signaling pathways such as MAPK, p53, NF-κB, and Nrf2 or through the execution of, for instance, caspases, B-cell lymphoma 2 (Bcl-2), or cytochrome c [84] (Figure 3).

### 4.1. Nrf2 Pathway

Most of the available information is related to the regulation of Nrf2 by miRNAs after irradiation. As already mentioned, if activated, this transcription factor regulates the synthesis of antioxidant enzymes and further cytoprotective molecules, with the aim to rescue the irradiated cell from apoptosis or other forms of cell death [85]. In melanoma cells, UV radiation can contribute to miRNA-206 modulation through the indirect activation of Nrf2 [86]. Overexpression of miRNA-630 triggers the activation of the Nrf2 molecule, subsequently promoting the upregulation of the antioxidant enzyme GPx2. This results in a reduction in cellular ROS levels, attenuating mitochondrial depolarization and diminishing radiation-induced DNA damage. These, in turn, decrease the intrinsic cellular apoptotic response in HNC (head and neck cancer) cell lines [87]. Qiu et al. [88] showed that activator protein 2a (AP2a) activated miRNA-125a-5p in HUVECs treated with X rays. This upregulation of miRNA-125a-5p ameliorated oxidative stress injury of HUVECs caused by radiation through Nrf2/HO-1 signaling. Cheng et al. [89] demonstrated that miRNA-141 directly targets Keap1 to activate Nrf2 signaling, which protects retinal pigment epithelium cells (RPEs) and retinal ganglion cells (RGCs) from UV radiation. UV-induced ROS production and cell death were significantly attenuated in miRNA-141-expressing RPEs and RGCs. On the other hand, the depletion of miRNA-141 when expressing its inhibitor antagomiR-141 led to Keap1 upregulation and Nrf2 degradation, which aggravated UV-induced death of RPEs and RGCs.

### 4.2. NF-κB Pathway

In response to radiation, NF-κB is known to reduce cell death by promoting the expression of anti-apoptotic proteins and the activation of the cellular antioxidant defense system [90]. The ability of UV to induce miRNA-21 overexpression in melanoma involves the ROS-mediated modulation of transcription factors such as the signal transducer and activator of transcription 3 (STAT3), activator protein 1 (AP-1), and NF-κB, which all have recognition sites on the miRNA-21 promoter [91]. Moreover, miRNA-21 upregulation was also related to the UV-mediated ROS induction of NF-κB and AP-1 activities [92]. Yang et al. [93] investigated the role of miRNA-4497 in oxidative stress and inflammatory injury in keratinocytes induced by UVB radiation. According to their results, UVB may promote the expression of inflammatory and oxidative stress signals in keratinocytes by upregulating miRNA-4497 expression, thus mediating cell injury. Downregulation of miRNA-4497 expression significantly inhibited the effects of UVB radiation on cell proliferation, apoptosis, cytokine secretion, the redox level, and the NF-κB signal in keratinocells.

It has been shown that radiation induces cell apoptosis through the upregulation of tumor necrosis factor alpha (TNF-α) [94]. The results of Kura et al. [95] showed that the levels of MDA and TNF-α increased in the rat myocardium after irradiation, while miRNA-1 and -15b were significantly decreased in the rat myocardium after irradiation. On the other hand, the authors observed a significant increase in miRNA-21 expression in the irradiated rat hearts. Zhang et al. [52] found that miRNA-21 inhibited the metabolism of superoxide to hydrogen peroxide, produced either via endogenous basal activities or exposure to ionizing radiation, directly by targeting SOD3 or through an indirect mechanism that limited TNF-α production, thereby reducing SOD2 levels in human bronchial epithelial cells.

### 4.3. p53 Pathway

In response to radiation-induced oxidative stress, p53 has an essential role in the regulation of the redox state. When intracellular ROS levels are extensively increased by radiation, p53 can be activated by c-Jun N-terminal kinase (JNK) signaling, which is responsible for the upregulation of pro-oxidant genes [96]. Moreover, it has been reported that p53 might be involved in the suppression of antioxidants associated with Nrf2 [97]. miRNA-125b might be induced by irradiation [78]. miRNA-125b regulates human lens epithelial cell apoptosis at least in part by directly targeting p53 [98]. miRNA-34a, a direct target of p53, can be induced by irradiation in different tissues and mice strains. After irradiation, miRNA-34a enhanced cell apoptosis and decreased cell viability in mice. miRNA-34a was correlated with tissue differences in radio-sensitivity. One possible downstream target of miRNA-34a that leads to a different radio-sensitivity is Bcl-2 [99].

### 4.4. FOXO Pathway

Transcription factors of the FOXO family are important regulators of the cellular stress response and promote the cellular antioxidant defense [100]. FOXO3a may effectively increase the cellular antioxidant capacity by enhancing the levels of CAT and PRX3 to protect against oxidative stress [101]. A previous study indicated that the depletion of FOXO3a expression profoundly reduced Keap1 protein levels, thereby activating Nrf2 signaling [102]. Similarly, FOXO1 induces the expression of antioxidant genes to decrease apoptosis [103]. Radiation can significantly increase the expression of miRNA-155-5p [104]. In human pancreatic cells, Wang et al. [105] revealed that miRNA-155 promotes ROS levels by directly targeting a transcription factor FOXO3a that induces SOD2 and CAT transcription. miRNA-27a was downregulated in response to UVB radiation in cutaneous squamous cell carcinoma cells [106]. Guttilla and White [107] found that in breast cancer cells, miRNA-27a directly targeted FOXO1. Overexpression of FOXO1 resulted in decreased cell viability and induction of cell death. miRNA-182 is responsive to irradiation. A previous study showed that miRNA-182 knockdown suppressed cell proliferation and increased cell apoptosis after irradiation in lung cancer cells. FOXO3 was proven to be a direct target of miRNA-182 [108].

### 4.5. SIRT Pathway

SIRTs are modulators in oxidative stress and inflammation, as they can regulate the expression and activation of downstream transcriptional factors (such as FOXO, Nrf2, and NF-κB) as well as antioxidant enzymes [109]. Joo et al. [110] demonstrated that SIRT1 regulates cell survival upon the induction of apoptotic stress in HeLa and 293T cells after irradiation. Irradiation regulates miRNA-34a by increasing its expression [111]. miRNA-34a upregulates p53 activity and functioning by downregulating SIRT1, which is a negative regulator of p53 through deacetylating p53 [112]. miRNA-200c-3p was significantly upregulated in human and mouse serum after irradiation [113]. It has been demonstrated that miRNA-200c directly targets SIRT1, eNOS, and FOXO1 in vitro as well as in vivo. This promoted ROS production and decreased NO, contributing to endothelial dysfunction under conditions of increased oxidative stress [48]. Sun et al. [114] showed that inhibition of miRNA-4532 protects human lens epithelial cells (HLECs) from UV-induced oxidative injury via activation of the SIRT6-Nrf2 pathway. Overexpression of miRNA-4532 in HLECs decreased SIRT6 activity, causing SIRT6 downregulation and Nrf2 signaling inhibition.

### 4.6. MAPK Pathway

The MAPK pathway transduces signals from the cell membrane to the nucleus in response to a variety of different stimuli. Multiple signal transduction pathways stimulated by ionizing radiation are mediated by the MAPK pathway, including those for the extracellular-signal-regulated kinase (ERK), JNK, and p38 MAPK. Activation of JNK and p38 MAPK by stress stimuli is strongly associated with apoptotic cell death [115]. A study in mesenchymal stem cells indicated that miRNA-21 enhances ROS production via the MAPK pathway and suppresses SOD2, SOD3, and sprouty homolog 2 (SPRY-2) expression to regulate the stem cell fate [116]. Li et al. [47] concluded that miRNA-182-5p inhibited lens epithelial cells’ apoptosis by regulating NOX4 and p38 MAPK signaling. A dual-luciferase reporter assay confirmed that NOX4 was a direct target that was downregulated by miRNA-182-5p. miRNA-21 and miRNA-182-5p expression was induced by ionizing radiation [117,118].

miRNAs may regulate ROS production or elimination by targeting various other redox-sensitive proteins. Irradiation induces miRNA-22 in many cell types, including bone marrow mesenchymal stromal cells, thereby increasing mitochondrial ROS and cellular apoptosis. Regulated in development and DNA damage responses 1 (Redd1) was found to be a direct target for miRNA-22, and overexpression of Redd1 diminished the role of miRNA-22 in mitochondrial ROS generation protecting cells from radiation-induced cell injury [119]. In another study, whole-heart irradiation with a single dose of 20 Gy significantly inhibited the expression of miRNA-223-3p in mice hearts. An miRNA-223-3p mimic significantly relieved—while an miRNA-223-3p inhibitor aggravated—apoptosis, oxidative damage, and cardiac dysfunction induced by irradiation. The protective effect of miRNA-223-3p was mediated by activating adenosine monophosphate-activated protein kinase (AMPK) [120]. de Souza et al. [121] investigated the effects of radiotherapy on the levels of hypoxic markers in oral squamous cell carcinoma. Radiotherapy reduced the levels of HIF-1α, miRNA-210, and lactate dehydrogenase (LDH) in vivo and in vitro.

## 5. miRNAs as Potential Therapeutics

miRNA therapeutics can be used to modify pathological miRNA expression changes. Synthetic miRNAs, called miRNA mimics, and miRNA inhibitors, called anti-miRs, are most often used to modify miRNA levels [122]. miRNA mimics mimic mature endogenous miRNAs with the aim to replace downregulated or missing miRNA expression. miRNA inhibitors that mimic miRNA target sequences can be used in the form of anti-miR to lower elevated levels of miRNAs [123]. Another option is offered by miRNA sponges, which are plasmid constructs that contain multiple miRNA binding sites. These transcripts can efficiently sequester specific miRNAs, preventing their binding to target genes [124].

miRNA drugs intended for clinical testing are mainly administered via the skin or intravenous injection, although administration by inhalation is also possible [125]. To overcome the low permeability of miRNAs through cellular membranes and to ensure a resistance to nuclease degradation, more efficient delivery can be achieved through the introduction of chemical modifications or conjugation to biomolecules. The use of nano-sized carrier vehicles can also enhance the uptake to cells [126]. Another strategy is to incorporate an miRNA into a carrier vehicle, e.g., a liposome [127]. Nonpathogenic viral vectors offer another option for intracellular delivery of miRNA-based therapeutics [125].

Some miRNA drugs have exhibited promising efficacy in a range of health conditions, including cancer, hepatitis C, heart abnormalities, and kidney disease [128]. However, up to now, only a few miRNA-based therapeutics have entered a clinical test phase [125]. Antagomir, targeting miRNA-122, was used to treat patients with chronic hepatitis C virus. Administration of anti-miR-122 resulted in a substantial viral load reduction in all treated patients within 4 weeks and a sustained virological response in three patients for 76 weeks. However, the trial was terminated due to some serious adverse effects [129]. Preclinical studies have shown that treatment with an anti-miR-21 significantly attenuates kidney failure by reducing the rate of progression of renal fibrosis [130]. A mimic of miRNA-29 reduces the expression of collagen and other proteins involved in scar formation, exhibiting inhibitory effects on fibrosis [131]. The administration of an miRNA-155 inhibitor led to the reduced growth of tumorigenic lymphoid cells in vivo, suggesting that targeting miRNA-155 is promising for treating leukemia and lymphoma [132]. In a clinical study involving patients with failing hearts, the administration of an miRNA-132 inhibitor normalized the electrocardiogram and produced positive trends for cardiac fibrosis markers [133].

On the other hand, there were also clinical studies where the use of miRNA drugs did not yield the expected results. In one, an miRNA-34a mimic was designed to treat several cancers. However, a phase I clinical trial in patients with solid tumors concluded with only 3 patients achieving sustained confirmed partial responses and 14 patients maintaining stable disease. Due to severe immune-mediated adverse events, the trial was terminated [127]. The results of another clinical study using a synthetic double-stranded mimic of miRNA-16 encapsulated by a bacterial minicell system were also not satisfying. Of the 22 patients with recurrent malignant pleural mesothelioma who were assessed for a therapeutic response, 1 had a partial response, 15 had stable disease, and 6 had progressive disease. Moreover, several dose-limiting toxicities were noted, mainly cardiovascular complications and lymphopenia [134].

As already mentioned, the action of miRNAs is highly complex, because each miRNA can act upon several different target genes, whilst one target gene can be regulated by many different miRNAs [5]. This is considered one of the main challenges in designing miRNA-based therapeutics as it may lead to off-target effects [135]. miRNA-34a acts as a tumor suppressor by regulating tumor suppressor genes like p53 [136]. In a clinical study with a synthetic miRNA-34a mimic for tumor treatment, severe immune-related side effects emerged, causing the death of four patients [127]. Immunogenicity has also been reported for the use of viral delivery systems [137]. The other obstacles in the use of miRNA drugs are practical difficulties, including the identification of effective administration routes, the control of in-body stability, the targeting of specific tissues and cell types, and the attaining of the intended intracellular effects [125].

## 6. Conclusions

People are exposed to radiation in their daily lives, e.g., during medical diagnosis and treatment, airport controls, UV radiation, and cosmic rays. There is increasing call for researchers to investigate the functional relationships between miRNA expression and irradiation. This review summarizes the importance of the link between miRNAs and oxidative stress in radiation-induced injury. During irradiation, miRNAs mainly act with ROS to regulate the cell fate. miRNAs have been shown to interfere with ROS-generating enzymes (NOX) as well as with antioxidant enzymes participating in their elimination (SOD, CAT, GPx). Modulation of transcription factors (Nrf2, NF-κB, p53, FOXO) and other redox-sensitive genes (MAPK, SIRT) by miRNAs has also been described. Among the miRNAs linked to oxidative stress induced by irradiation, miRNA-21 and miRNA-34a are the most intensively studied, although many other miRNAs have been identified to be involved in radiation-induced oxidative stress, e.g., miRNA-124, miRNA-125a, miRNA-141, miRNA-155, etc. In this context, the Nrf2 and NF-κB signaling pathways were the most often investigated. The available information supports the existence of a complex crosstalk between miRNAs and ROS induced by irradiation. The results of several studies indicate that miRNAs may play an important role in the prevention and treatment of cardiovascular diseases induced by oxidative stress not only from irradiation but also from other sources. These findings may help to develop novel therapeutic strategies for treating radiation-induced oxidative stress injury.

## Figures and Tables

**Table 1:** Examples of miRNAs directly targeting ROS-producing enzymes. NADPH oxidase (NOX)-, endothelial nitric oxide synthase (eNOS), human umbilical vein cells (HUVECs), ischemia–reperfusion (I/R).

miRNA	ROS-Producing Enzyme	Experimental Model	Reference
miRNA-124-5p	NOX2	rats cerebral I/R injury	[42]
miRNA-34a	NOX2	human glioma cells	[43]
miRNA-320	NOX2	ischemic cerebral neurons	[44]
miRNA-423-5p	NOX4	mouse podocyte cells	[45]
miRNA-99a	NOX4	lung adenocarcinoma cells	[46]
miRNA-182-5p	NOX4	human lens epithelial cells	[47]
miRNA-200c	eNOS	HUVEC cells	[48]
miRNA-615-5p	eNOS	HUVEC cells	[49]
miRNA-155	eNOS	HUVEC cells	[50]

**Table 2:** Examples of miRNAs directly targeting endogenous antioxidant enzymes. Superoxide dismutase (SOD), glutathione peroxidase (GPx), thioredoxin reductase (TXNRD), catalase (CAT), glutathione reductase (GR).

miRNA	Antioxidant Enzyme	Experimental Model	Reference
miRNA-21	SOD	human bronchial epithelial cells	[52]
miRNA-17-3p	SOD, GPx, TXNRD	prostate cancer cells	[53]
miRNA-23a	SOD	cardiomyocytes	[54]
miRNA-30b	CAT	human retinal pigment epithelial cell line	[55]
miRNA-551b	CAT	human lung cancer cells	[56]
miRNA-144	GPx, GR	human neuroblastoma SH-SY5Y cells	[57]
miRNA-181a	GPx	cardiomyocytes	[58]
miRNA-214	GR	liver cells	[59]

## Abbreviations

4-HNE	4-hydroxy-2-nonenal
8-OHdG	8-hydroxy-2′-deoxyguanosine
AKT	protein kinase B
AMPK	adenosine monophosphate-activated protein kinase
AP-1	activator protein 1
AP2a	activator protein 2a
Bcl-2	B-cell lymphoma 2
CAT	catalase
COX	cyclooxygenase
DSB	double-strand break
eNOS	endothelial nitric oxide synthase
ERK	extracellular-signal-regulated kinase
FOXO	Forkhead box O
GPx	glutathione peroxidase
GR	glutathione reductase
HIF-1α	hypoxia-inducible factor 1 alpha
HLEC	human lens epithelial cell
HO	heme oxygenase
H_2_O_2_	hydrogen peroxide
HUVEC	human umbilical vein cell
IκBα	inhibitory protein alpha of nuclear factor kappa B
JNK	c-Jun N-terminal kinase
Keap 1	Kelch-like ECH-associated protein 1
LDH	lactate dehydrogenase
LOX	lipoxygenase
MAPK	mitogen-activated protein kinase
MDA	malondialdehyde
mRNA	messenger RNA
miRNA	microRNA
NF-κB	nuclear factor kappa B
NOS	nitric oxide synthase
NOX	NADPH oxidase
Nrf2	nuclear factor erythroid 2-related factor 2
O_2_^•−^	superoxide anion
^•^OH	hydroxyl radical
p53	tumor suppressor protein p53
PRDX	peroxiredoxin
pre-miRNA	precursor form of microRNA
pri-miRNA	primary nuclear transcript of microRNA
Redd1	regulated in development and DNA damage responses 1
RGC	retinal ganglion cell
RISC	RNA-induced silencing complex
RNS	reactive nitrogen species
ROS	reactive oxygen species
RPE	retinal pigment epithelium cells
SIRT	sirtuin
SOD	superoxide dismutase
SPRY-2	sprouty homolog 2
SSB	single-strand break
STAT3	signal transducer and activator of transcription 3
TXN	thioredoxin
TXNRD	thioredoxin reductase
